# Direct but Not Indirect Methods Correlate the Percentages of Sperm With Altered Chromatin to the Intensity of Chromatin Damage

**DOI:** 10.3389/fvets.2021.719319

**Published:** 2021-08-25

**Authors:** Jordi Ribas-Maynou, Marc Llavanera, Yentel Mateo-Otero, Estela Garcia-Bonavila, Ariadna Delgado-Bermúdez, Marc Yeste

**Affiliations:** ^1^Biotechnology of Animal and Human Reproduction (TechnoSperm), Institute of Food and Agricultural Technology, University of Girona, Girona, Spain; ^2^Unit of Cell Biology, Department of Biology, Faculty of Sciences, University of Girona, Girona, Spain

**Keywords:** sperm, chromatin, DNA fragmentation, TUNEL, SCSA, comet assay

## Abstract

Although sperm chromatin damage, understood as damage to DNA or affectations in sperm protamination, has been proposed as a biomarker for sperm quality in both humans and livestock, the low incidence found in some animals raises concerns about its potential value. In this context, as separate methods measure different facets of chromatin damage, their comparison is of vital importance. This work aims at analyzing eight techniques assessing chromatin damage in pig sperm. With this purpose, cryopreserved sperm samples from 16 boars were evaluated through the following assays: TUNEL, TUNEL with decondensation, SCSA, alkaline and neutral sperm chromatin dispersion (SCD) tests, alkaline and neutral Comet assays, and chromomycin A3 test (CMA3). In all cases, the extent of chromatin damage and the percentage of sperm with fragmented DNA were determined. The degree of chromatin damage and the percentage of sperm with fragmented DNA were significantly correlated (*p* < 0.05) in direct methods (TUNEL, TUNEL with decondensation, and alkaline and neutral Comet) and CMA3, but not in the indirect ones (SCD and SCSA). Percentages of sperm with fragmented DNA determined by alkaline Comet were significantly (*p* < 0.05) correlated with TUNEL following decondensation and CMA3; those determined by neutral Comet were correlated with the percentage of High DNA Stainability (SCSA); those determined by SCSA were correlated with neutral and alkaline SCD; and those determined by neutral SCD were correlated with alkaline SCD. While, in pigs, percentages of sperm with fragmented DNA are directly related to the extent of chromatin damage when direct methods are used, this is not the case for indirect techniques. Thus, the results obtained herein differ from those reported for humans in which TUNEL, SCSA, alkaline SCD, and alkaline Comet were found to be correlated. These findings may shed some light on the interpretation of these tests and provide some clues for the standardization of chromatin damage methods.

## Introduction

The research of biomarkers that predict sperm fertilizing ability has gained much interest in the last years and has led to the discovery of factors affecting reproductive outcomes in both humans and production animals (1–4). In humans, infertility affects millions of couples worldwide and has an incidence of ~7–15%, being a multifactorial disease due to both male and female factors (5). As clinical treatments for infertile patients usually involve *in vitro* fertilization (IVF) and intracytoplasmic sperm injection (ICSI) and pregnancy rates using these methods are relatively low (6), research on predicting IVF/ICSI outcomes is much warranted. Contrastingly, in livestock animals, reproductive performance has been improved after many years of genetic selection of the best breeders (7). In pigs, artificial insemination is the most used method for breeding, and the quality of liquid-stored and cryopreserved sperm is evaluated to determine the potential fertility of males (8, 9). In this context, finding sperm quality biomarkers that allow the selection of the most suitable boars is crucial to increase reproductive efficiency in farms (10–12).

In the last decades, testing sperm chromatin defects has turned into a reliable biomarker of seminal quality in humans and animals (13, 14). As sperm DNA breaks are known to be important for the diagnosis of male infertility in humans (15), few reports were focused on elucidating whether or not this sperm quality parameter could predict the reproductive efficiency of production animals (16–19). DNA fragmentation can be analyzed directly, using techniques such as TUNEL and Single Cell Gel Electrophoresis (also known as Comet assay), or indirectly, through methods like Sperm Chromatin Structure Assay (SCSA) and Sperm Chromatin Dispersion test (SCD test or halo assay). These indirect techniques determine the amount of DNA damage through the differential chromatin decondensation of fragmented DNA (2, 14, 20).

The utility of chromatin fragmentation has been the source of much debate, as whereas some studies point out to the detrimental effect of DNA damage on assisted reproduction techniques (21, 22), others find opposite or inconclusive results (23–25). The reason of this controversy is related to the differences in both the methods for measuring DNA damage and the clinical outcomes used to compare these methods (26). Remarkably, while the techniques evaluating sperm DNA fragmentation in farm animals have not been compared, only four studies contrasted different DNA fragmentation methods in humans, focusing on the male infertility condition and IVF/ICSI outcomes (27–30), and one analyzed the matter in mice (31). All these reports agreed in establishing correlations between TUNEL and alkaline Comet methods, and suggested that Comet would be one of the most sensitive methods to assess sperm DNA fragmentation (32). While several efforts to obtain standardized results among laboratories have been made, controversies remain open regarding the correlation between direct (e.g., Comet and TUNEL) and indirect (e.g., SCSA and SCD) assays, as results differ between studies (27, 28). Apart from the lack of research about the correlation between techniques in livestock sperm, most studies in pigs used indirect methods, like SCD or SCSA, to assess the relationship between DNA damage and fertilizing ability (18, 19, 33, 34).

Therefore, the aim of the present study was to compare different methods (direct and indirect) that evaluate sperm DNA fragmentation in pigs, in order to establish whether these tests are correlated with each other regarding both the percentage of sperm with fragmented DNA and the incidence of chromatin damage.

## Materials and Methods

### Reagents

Unless stated otherwise, all reagents were purchased from Sigma-Aldrich (St. Louis, MO, USA).

### Semen Samples

Sixteen semen samples collected from sexually mature boars (18–30 months of age) were provided by a local farm that operates under standard commercial conditions (Servicios Genéticos Porcinos S.L.; Roda de Ter, Spain). Animals were not directly manipulated for the present study; thus, it did not require any specific ethical approval.

An ejaculate from each boar was obtained using the gloved-hand method, and samples were immediately diluted 1:2 (v/v) in a long-term extender (Vitasem, Magapor S.L.; Zaragoza, Spain). Just after arrival, an aliquot was intended to evaluate sperm motility and viability; the remaining volume was cryopreserved following the standard protocol utilized before in our research group (35, 36).

### Sperm Cryopreservation

Semen samples were split into 50-ml aliquots, centrifuged at 2,400 × *g* and 17°C for 4 min, resuspended to a concentration of 1.5 × 10^9^ sperm/ml in β-Lactose-egg yolk (LEY) media (80% v/v lactose, 20% v/v egg yolk), and cooled down to 5°C at a rate of −0.1°C/min. Afterwards, samples were diluted in LEYGO medium [LEY medium supplemented with 6% v/v glycerol and 1.5% Orvus ES Paste (Equex STM; Nova Chemical Sales Inc., Scituate, MA, USA)] to a final concentration of 1 × 10^9^ sperm/ml. Finally, samples were loaded into 0.5-ml straws (MiniTüb; Tiefenbach, Germany) and submitted to the following curve: 6°C/min from 5 to −5°C (100 s); −39.82°C/min from −5 to −80°C (113 s); holding at −80°C for 30 s; −60°C/min from −80 to −150°C (70 s). At this point, samples were stored in liquid nitrogen until used.

Thawing was carried out by immersion of straws in a water bath at 38°C for 15 s, with shaking. The content of each straw was diluted with three volumes of Beltsville Thawing Solution (BTS) (0.2 M glucose, 23 mM sodium citrate, 15 mM sodium bicarbonate, 4.2 mM EDTA, 10 mM potassium chloride, and 0.1 mM kanamycin, pH 7.5).

### Sperm Motility Analysis Through Computer-Assisted Sperm Analysis

A commercial computer-assisted system (Integrated Sperm Analysis System V1.0; Proiser S.L., Valencia, Spain) was used to analyze sperm motility before and after cryopreservation. First, samples were warmed at 38°C for 15 min, and 5 μl was loaded onto a previously warmed (38°C) Makler chamber (Sefi-medical Instruments, Haifa, Israel). Different fields were captured at 100 × magnification, recording 25 consecutive images at 25 images per second, until reaching 1,000 spermatozoa per assessment. At least two technical replicates per sample were evaluated, and the mean of replicates was recorded. The following parameters were assessed: percentage of total motile sperm; percentage of progressive motility; percentages of sperm with rapid, medium, and slow motility; curvilinear velocity (VCL; the instantaneously recorded sequential sperm progression along the whole trajectory; μm/s); straight-line velocity (VSL; the straight sperm trajectory per unit of time; μm/s); average path velocity (VAP; the mean sperm trajectory per unit of time; μm/s); linearity coefficient (LIN = VSL/VCL × 100; %); straightness coefficient (STR = VSL/VAP × 100; %); wobble coefficient (WOB: VAP/VCL × 100; %); mean amplitude of lateral head displacement (ALH; the mean amplitude of the lateral oscillatory movement of the sperm head around the mean trajectory; μm); and frequency of head displacement (BCF; the number of sperm head lateral oscillatory movements around the mean trajectory per unit of time; Hz).

### Evaluation of Sperm Morphology

In order to assess sperm morphology, samples were incubated in 2% formaldehyde at room temperature for 5 min. Sperm cells were analyzed through SCA production Software (Sperm Class Analyzer Production, 2010; Microptic S.L., Barcelona) and classified as morphologically normal, with proximal or distal droplets, or aberrant (including head and tail anomalies). Three replicates (100 sperm each) per sample were counted.

### Determination of Sperm Viability Through Flow Cytometry

Sperm viability was evaluated by assessing plasma membrane integrity with LIVE/DEAD Sperm Viability Kit (Molecular Probes, Eugene, OR, USA). Sperm diluted at a final concentration of 1 × 10^6^ in pre-warmed PBS were first incubated with SYBR14, a membrane-permeable fluorochrome that stains sperm heads in green, for 10 min (final concentration: 32 nmol/L). Thereafter, samples were incubated with propidium iodide (PI), a membrane-impermeable fluorochrome that only penetrates membrane-damaged sperm, for 5 min (final concentration: 7.5 μmol/L).

Stained sperm were examined through a CytoFLEX cytometer (Beckman Coulter; Fullerton, CA, USA). Samples were excited with an argon-ion laser at 488 nm and 10,000 events per replicate were evaluated, using FITC (BP 525/40) and PC5.5 (690/50) filters for SYBR14 and PI, respectively. Three separate populations were identified, a membrane-intact sperm population (SYBR14^+^/PI^−^) and two subpopulations of membrane-damaged sperm with different degrees of alteration (SYBR14^+^/PI^+^ and SYBR14^−^/PI^+^). Three technical replicates per sample were examined, and data were not compensated.

### Neutral and Alkaline Comet Assay

Sperm Comet assay performed in neutral pH conditions was used to determine double-strand DNA breaks, whereas alkaline Comet assay was conducted to assess the total amount of DNA damage consisting of single- and double-strand DNA breaks. The Comet assay was based on the protocol described by Ribas-Maynou et al. (37) for pig sperm, which completely decondenses DNA prior to conducting the test.

#### Sperm Fixation and Lysis

Sperm samples were first diluted to 5 × 10^5^ spermatozoa/ml in PBS, and mixed 1:2 (v/v) with melted low melting point agarose at 37°C. Two 6.5-μl drops of the mixture were poured onto two agarose-pretreated slides—one for alkaline Comet and the other for neutral Comet—which were subsequently covered with an 8-mm-diameter round coverslip. Then, agarose–sample mixtures were allowed to jellify on a cold plate at 4°C, coverslips were gently removed, and both slides were incubated in three lysis solutions (all at pH 7.5): (1) 0.8 M Tris-HCl, 0.8 M DTT, and 1% SDS for 30 min; (2) 0.8 M Tris-HCl, 0.8 M DTT, and 1% SDS for 30 min; and (3) 0.4 M Tris-HCl, 0.4 M DTT, 50 mM EDTA, 2 M NaCl, 1% Tween20, and 100 μg/ml Proteinase K for 180 min.

#### Electrophoresis

Electrophoresis conditions differed between the two Comet variants. For slides designated to neutral Comet, electrophoresis was performed in TBE buffer (0.445 M Tris-HCl, 0.445 M Boric acid, and 0.01 M EDTA; pH 8) at 1 V/cm for 30 min; slides were subsequently washed in 0.9% NaCl solution for 2 min.

Slides designated to alkaline Comet were denatured in cold alkaline solution (0.03 M NaOH and 1 M NaCl) for 5 min and then electrophoresed at 1 V/cm for 4 min in an alkaline buffer (0.03 M NaOH; pH 13).

#### Neutralization, Dehydration, and Staining

After electrophoresis, both slides were incubated in neutralizing solution (0.4 M Tris-HCl; pH 7.5), dehydrated in ethanol series (70, 90, and 100%), and dried in horizontal position. Staining was conducted with 5 μl of 1 × Safeview DNA stain (NBS Biologicals, Huntingdon, UK) and covered with 20 × 20 coverslips.

#### Imaging and Analysis

Comets were observed under an epifluorescence microscope (Zeiss Imager Z1, Carl Zeiss AG, Oberkochen, Germany). Captures of at least 100 cells per sample were performed at 100 × magnification and at a resolution of 1,388 × 1,040 pixels using Axiovision 4.6 software (Carl Zeiss AG, Oberkochen, Germany). Exposure time was adjusted to avoid overexposure of Comet heads.

Analysis of both neutral and alkaline Comets was performed using the open-access CometScore v2.0 software (Rexhoover, www.rexhoover.com), which analyzes the fluorescence intensity of Comet heads and tails. After automatic analysis, a manual review of the analyzed Comets was performed to eliminate captures that did not correspond to Comets or tallied with the overlapping ones. Furthermore, incorrect interpretation of the center of Comet heads due to misanalysis was corrected during manual revision. When the final number of correctly analyzed Comets was < 50, more captures until this figure was reached were performed.

For the quantitative analysis of the amount of DNA breaks (chromatin damage intensity), Olive Tail Moment (OTM), calculated as (Tail mean intensity–Head mean intensity) × Tail DNA/100, was chosen as a reference parameter (38).

#### Calculation of the Percentages of Damaged Sperm

Altered and normal sperm subpopulations were determined on the basis of the percentages of sperm with fragmented DNA in all samples. Tail Length, Tail DNA, and OTM were used to run a Principal Component Analysis (PCA). These parameters were sorted into one PCA component, and the obtained data matrix was rotated through the Varimax procedure with Kaiser normalization. Variables with a loading factor higher than 0.6 and lower than 0.3 in the rotated matrix were selected. The resulting component was used to calculate regression scores that were assigned to each spermatozoon. Regression scores were used to classify each Comet through a cluster analysis, using the between-groups linkage method based on the Euclidean distance. A total of three subpopulations were identified for both neutral and alkaline Comet assays. These subpopulations corresponded to sperm with high, medium or low amount of DNA breaks, respectively.

### Alkaline and Neutral Chromatin Dispersion Assay

Upon thawing, samples intended to alkaline and neutral SCD assays were washed twice in PBS (centrifugation at 600 × *g* for 5 min) and diluted to 2 × 10^6^ sperm/ml with PBS. Afterwards, samples were mixed (1:2) with low melting point agarose at 37°C; two drops of 6.5 μl of the mixture were allowed to jellify onto two agarose-pretreated slides at 4°C for 5 min and then covered with an 8-mm coverslip. At this point, the slide designated to alkaline variant was subjected to acid denaturation in 0.08 N HCl (39). Both slides were incubated in lysis solutions that allowed complete chromatin decondensation (37): 30 min for Lysis 1, 30 min for Lysis 2, and 180 min for Lysis 3. After lysis, slides were washed in distilled water, neutralized in neutralization solution (0.4 M Tris-HCl; pH 7.5), dehydrated in an ethanol series (70, 90, and 100%), and dried in horizontal position.

Analysis of the halo diameter was used as a quantitative parameter for DNA breaks (chromatin damage intensity), whereas the percentage of sperm with fragmented DNA was determined by classifying 400 sperm haloes using the criteria defined for sperm DNA fragmentation (40).

### Conventional TUNEL Assay and TUNEL Assay With Previous DNA Decondensation

TUNEL assay was performed in two variants: with previous decondensation and without decondensation. For both variants, the standard TUNEL protocol was applied according to the *In situ* Cell Death Detection Kit (Roche Diagnostic Gmbh, Penzberg, Germany).

First, samples were thawed and washed twice in PBS (centrifugation at 600 × *g* for 5 min). For the procedure that included decondensation, samples were incubated in 2 mM DTT at room temperature for 45 min (41) and then washed twice in PBS (centrifugation at 600 × *g* for 5 min). Afterwards, and for both variants, samples were resuspended in 300 μl of permeabilization solution (0.1% sodium citrate and 0.25% Triton X-100), incubated at 4°C for 2 min, and washed twice in PBS. In the second wash, samples were split into two tubes; pellets were resuspended in (a) 50 μl of labeling solution (negative control) and (b) 50 μl of TUNEL reaction mixture (containing 45 μl of labeling solution and 5 μl of terminal deoxynucleotidyl transferase enzyme). Samples were incubated at 37°C for 60 min and subsequently washed twice in PBS. Finally, samples were diluted to a final volume of 500 μl and analyzed by flow cytometry. A positive control, which consisted of incubating the sample with 4 units/μl of DNAse I (ThermoFisher Scientific, Waltham, USA) supplemented with 10 mM MgCl_2_ at 37°C for 1 h, was performed for setting the technique up.

A total of 10,000 spermatozoa were analyzed with a CytoFLEX flow cytometer (Beckman Coulter, Fullerton, CA, USA), using the FITC channel, with an excitation wavelength of 488 nm and a detection wavelength of 525/40 nm. Data were processed using CytExpert software (Beckman Coulter, Fullerton, CA, USA), and the respective negative control of each sample was used to set up the threshold for TUNEL^+^ sperm (sperm with fragmented DNA). Quantitative analysis of DNA breaks was provided by the median of FITC intensity.

### Sperm Chromatin Structure Assay

Sperm samples were also evaluated through the standard SCSA procedure. Briefly, thawed samples were centrifuged twice at 600 × *g* for 5 min and resuspended with TNE buffer (10 mM Tris-HCl, 150 mM NaCl, and 1 mM EDTA; pH 7.5) to a final concentration of 2 × 10^6^ sperm/ml. Two hundred microliters of the sample were mixed with 400 μl of acid detergent solution (0.08 M HCl, 0.15 M NaCl, and 0.1% Triton X-100; pH 1.2). After 30 s, sperm were stained with Acridine Orange Staining solution (6 μg/ml Acridine Orange in buffer containing 0.037 M citric acid, 0.126 M Na_2_HPO_4_, 1.1 mM EDTA, and 0.15 M NaCl; pH 6.0) for 3 min. Five thousand sperm were analyzed through a Cell Laboratory QuantaSC cytometer (Beckman Coulter; Fullerton, CA, USA). Green and red fluorescence were collected through FL1 (BP: Dichroic/Splitter, DRLP: 550 nm, BP: 525 nm) and FL3 (LP: 670 nm), respectively. Data were not compensated.

Percentages of sperm DNA fragmentation (%SDF) were determined as the number of sperm cells with increased red fluorescence compared to the main population showing equilibrated red/green fluorescence. Percentages of high DNA stainability (%HDS) were calculated as the number of sperm cells with increased green fluorescence compared to the main population. The degree of chromatin damage was determined through the geometric mean of red fluorescence intensity (FL3).

### Chromomycin A3 Test

Chromomycin A3 (CMA3) competes with protamines for the binding to the minor groove of DNA. Briefly, after thawing, sperm were washed twice in McIlvine buffer (30 mM citric acid, 140 mM Na_2_HPO_4_, and 10 mM MgCl_2_) and diluted to 20 × 10^6^ sperm/ml. Stock solution of CMA3 was prepared at 500 μg/ml. Staining was performed in 12.5 μg/ml CMA3 at room temperature for 20 min. A negative control (without the addition of CMA3) for each sample was included. Afterwards, samples were diluted 1:10 (v:v) in filtered PBS and analyzed with a CytoFLEX flow cytometer (Beckman Coulter, Fullerton, CA, USA). CMA3 was excited with a 405-nm laser and its emission was acquired through the Violet 610 channel (610/20). Data were processed using CytExpert software (Beckman Coulter, Fullerton, CA, USA), and each negative control was used to establish the threshold for CMA3^+^ sperm. Quantification of aberrant protamination was based on the median intensity of the Violet610 channel. A positive control was performed for setting the technique up, consisting of incubating the sample in 5 mM DTT (ThermoFisher Scientific, Waltham, USA) at 37°C for 45 min.

### Statistical Analyses

Data were analyzed using the Statistics Package for Social Sciences ver. 25.0 (IBM Corp.; Armonk, NY, USA). Graphs were elaborated with GraphPad Prism 8.0 Software (GraphPad, San Diego, USA).

Normal distribution was determined with Shapiro-Wilk test and homogeneity of variances was tested with Levene test. As, even after linear transformation with log(x), √x, and arcsin √x, data did not fit with parametric assumptions, differences in sperm motility and viability before and after cryopreservation were determined through the Wilcoxon test. Correlations were analyzed through the non-parametric Spearman test. For all tests, the level of significance was set at *p* ≤ 0.05.

## Results

### Sperm Motility and Viability

Sperm motility and viability measured before and after cryopreservation are shown in Table 1. As expected, sperm motility parameters decreased after cryopreservation; specifically, progressive motility (*p* = 0.001) was reduced, whereas non-progressive motility (*p* = 0.01) and proportions of slow and static sperm increased (*p* = 0.002 and *p* < 0.001). Similarly, sperm viability was reduced after cryopreservation (*p* < 0.001).

**Table 1:** Sperm motility, viability, and morphology in fresh and frozen-thawed sperm samples.

	**Fresh samples**					**30 min post-thaw**					
	**Mean**		**Standard deviation**	**Median**	**Rank**	**Mean**		**Standard deviation**	**Median**	**Rank**	***P*-value**
Progressive motility (%)	69.46%	±	5.43%	69.75%	(21.95%)	51.10%	±	15%	54.14%	(50.15%)	0.001*
Non-progressive motility (%)	28.56%	±	4.43%	28.23%	(15.17%)	33.29%	±	8%	30.99%	(25.59%)	0.010*
Rapid velocity (%)	62.84%	±	20.12%	55.70%	(59.29%)	44.27%	±	16%	44.67%	(56.92%)	0.121
Medium velocity (%)	25.86%	±	12.96%	30.08%	(37.69%)	20.55%	±	5%	19.86%	(22.70%)	0.134
Slow velocity (%)	9.32%	±	7.42%	8.97%	(26.49%)	19.57%	±	7%	19.37%	(25.63%)	0.002*
Static sperm (%)	1.98%	±	2.00%	1.43%	(7.84%)	15.61%	±	12%	13.74%	(38.91%)	<0.001*
Circular tracks (*n*)	1,176.63	±	611.67	1,080.17	(2,191.00)	1,069.67	±	518.45	1,073.67	(1,819.00)	0.301
VCL (μm/s)	59.49	±	17.26	51.03	(51.03)	51.60	±	8.18	50.93	(32.30)	0.438
VSL (μm/s)	28.51	±	5.63	26.96	(21.17)	28.05	±	5.81	27.89	(15.96)	1.000
VAP (μm/s)	43.12	±	10.25	38.03	(30.65)	39.33	±	6.62	39.44	(23.90)	0.408
LIN (%)	49.70	±	8.58	51.32	(26.72)	54.25	±	7.23	54.59	(26.64)	0.030*
STR (%)	66.87	±	6.23	67.95	(19.23)	70.93	±	5.96	71.61	(19.25)	0.020*
WOB (%)	73.73	±	6.29	76.27	(19.51)	76.14	±	4.15	76.24	(17.50)	0.121
ALH (μm)	2.62	±	0.62	2.42	(2.10)	2.42	±	0.25	2.39	(0.84)	0.535
BCF (Hz)	6.38	±	0.29	6.48	(1.03)	6.12	±	0.65	6.41	(1.92)	0.255
Viability (% viable sperm)	89.17	±	5.84	89.68	(24.50)	51.23	±	12.67	53.25	(42.75)	<0.001*
Morphology (% normal morphology)	90.05	±	6.17	91.30	(25.40)						

*Asterisks indicate significant differences (p < 0.05) between fresh and frozen-thawed sperm (30 min post-thaw)*.

### Sperm Chromatin Damage and Correlation Between Chromatin Damage Intensity and Percentage of Affected Cells

Table 2 shows sperm chromatin status analyzed through eight separate methods. Table 2A shows the intensity of DNA damage measured through TUNEL, SCD, SCSA, and Comet assays, and that of chromatin regions with abnormal protamination evaluated through the CMA3 test. Table 2B depicts the percentage of affected cells above the damage threshold established for each technique.

**Table 2:** Sperm chromatin status analyzed through eight methods. (A) Intensity of damage. (B) Percentage of sperm with fragmented DNA or altered chromatin.

	**Mean**		**Standard Deviation**	**Median**	**Rank**
**A**					
TUNEL (FITC intensity, A.U.)	444.8	±	317.9	413.1	(958.3)
TUNEL decondensed (FITC intensity)	1,718.3	±	621.0	1,631.7	(2,142.8)
Neutral SCD (Halo area, pixels)	1,441.4	±	338.4	1,313.0	(1,049.6)
Alkaline SCD (Halo area, pixels)	1,400.3	±	455.4	1,219.6	(1,775.2)
CMA3 (Intensity 610 nm. A.U.)	333.0	±	65.6	332.1	(247.6)
SCSA (FL3 intensity, A.U.)	159.6	±	78.9	150.3	(274.3)
Alkaline comet (Olive tail moment)	14.6	±	3.2	14.6	(10.8)
Neutral comet (Olive tail moment)	4.1	±	2.0	4.2	(5.82)
**B**					
TUNEL (%SDF)	2.0%	±	2.0	1.9%	(6.9)
TUNEL decondensed (%SDF)	8.9%	±	5.8	9.1%	(21.7)
CMA3 (%positive cells)	11.59%	±	5.5	10.5%	(20.0)
Neutral SCD (%SDF)	2.4%	±	0.9	2.2%	(3.2)
Alkaline SCD (%SDF)	2.6%	±	2.6	2.1%	(8.3)
SCSA (%SDF)	2.6%	±	1.8	2.6%	(6.3)
SCSA (%HDS)	4.4%	±	2.8	4.81%	(9.6)
Alkaline comet (%highly damaged)	38.0%	±	20.6	33.6%	(68.3)
Alkaline comet (%medium damaged)	41.3%	±	15.9	44.3%	(59.7)
Alkaline comet (%low damaged)	20.7%	±	15.8	16.3%	(54.5)
Neutral comet (%highly damaged)	1.8%	±	2.1	1.2%	(6.9)
Neutral comet (%medium damaged)	45.5%	±	22.4	50.2%	(71.6)
Neutral comet (%low damaged)	52.7%	±	22.7	48.5%	(74.2)

Intensity of DNA damage (FITC intensity) correlated with the percentage of sperm with fragmented DNA in both the conventional TUNEL assay (*R*s = 0.764; *p* = 0.001) and TUNEL with DTT decondensation (*R*s = 0.659; *p* = 0.006). For alkaline Comet, the intensity of DNA damage (measured as OTM) correlated with percentages of high (*R*s = 0.792; *p* = 0.001) and low damage (*R*s = −0.782 *p* < 0.001). For neutral Comet, the intensity of double-stranded DNA damage (measured as OTM) correlated with the percentage of low damage (*R*s = −0.559; *p* = 0.024). For CMA3, the intensity of abnormal protamination also correlated with the percentage of altered sperm (*R*s = 0.779; *p* < 0.001).

In contrast, no correlation between intensity and percentage of affected cells in alkaline SCD (*R*s = 0.263; *p* = 0.326), neutral SCD (*R*s = 0.024; *p* = 0.931), and SCSA (*R*s = 0.241; *p* = 0.368) was found.

### Correlations Regarding Chromatin Damage Intensity Between Tests

**Figure 1:**
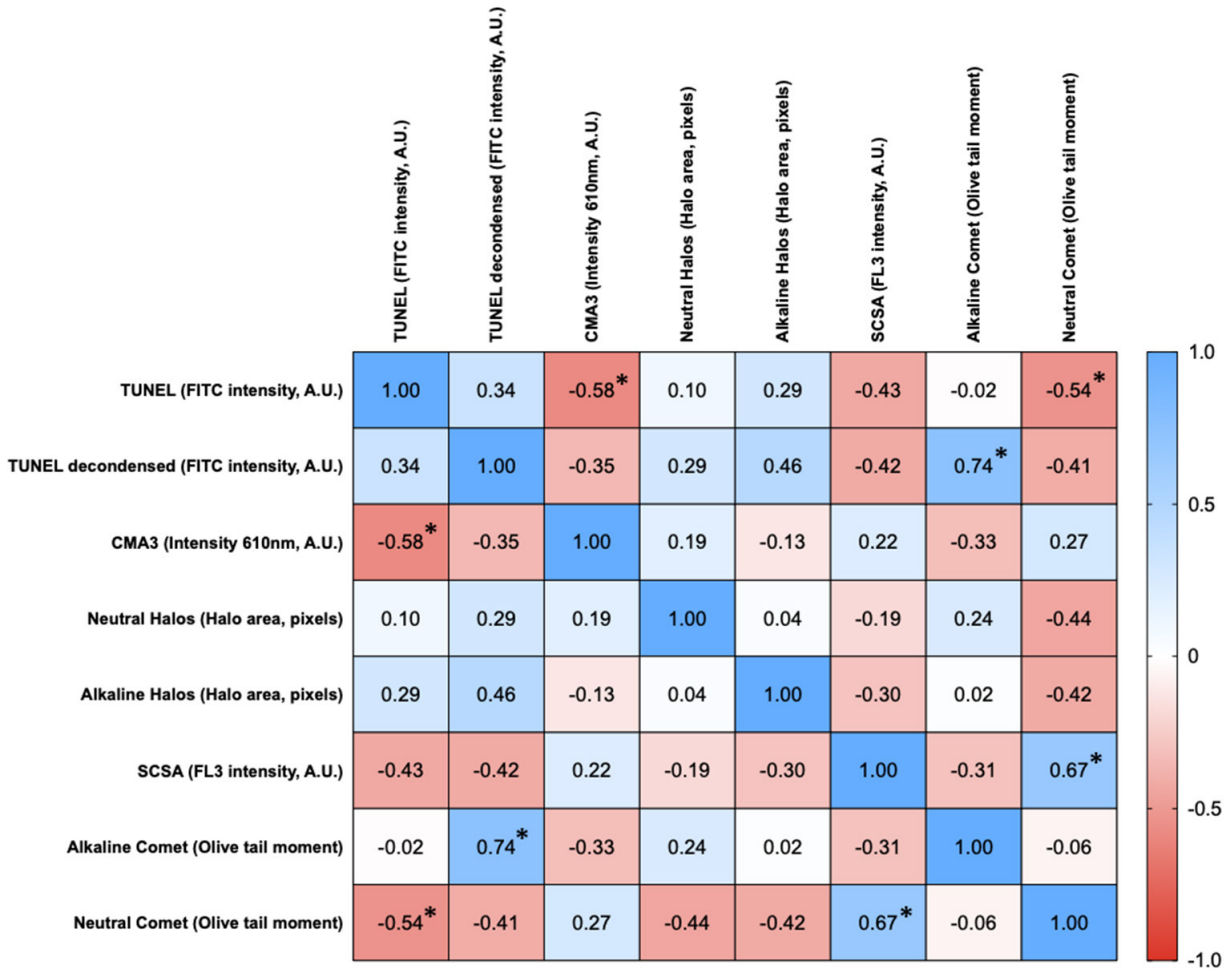
Correlation matrix between methods regarding the degree of sperm chromatin damage. Figures in each box represent Spearman correlation coefficients. Asterisks and bold figures indicate statistically significant correlations (*p* < 0.05).

**Figure 2:**
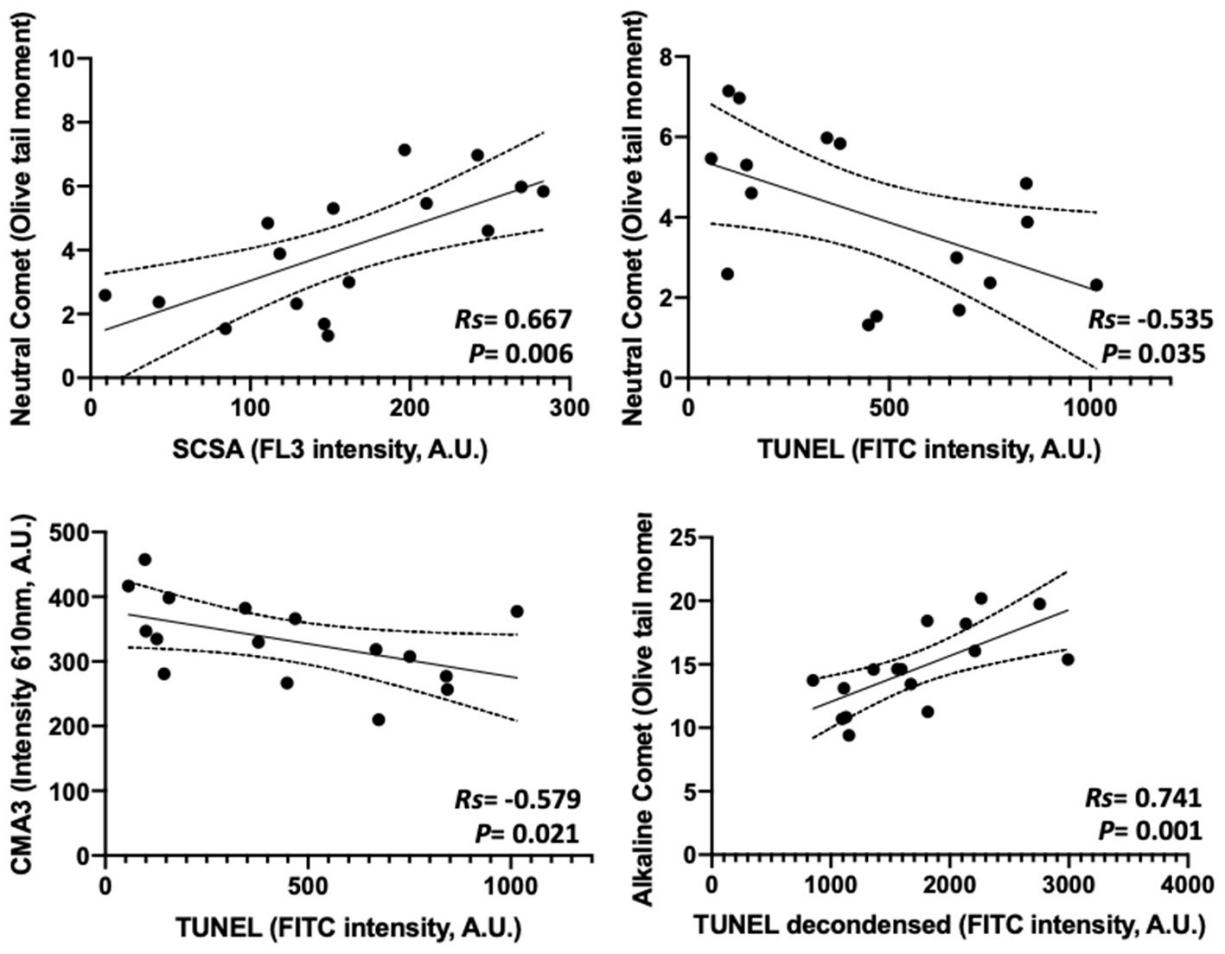
Statistically significant correlations between parameters analyzing the degree of chromatin damage and obtained through different methods.

Figure 1 shows the matrix of correlations between different chromatin damage methods regarding damage intensity. Statistically significant correlations were found between neutral Comet OTM and SCSA (*R*s = 0.667; *p* = 0.006), between neutral Comet OTM and TUNEL (*R*s = −0.535; *p* = 0.035), between conventional TUNEL and CMA3 (*R*s = −0.579; *p* = 0.021), and between alkaline Comet OTM and TUNEL after decondensation (*R*s = 0.741; *p* = 0.001). Figure 2 represents these statistically significant correlations.

### Correlations Between Chromatin Damage Methods Regarding Percentage of Affected Cells

**Figure 3:**
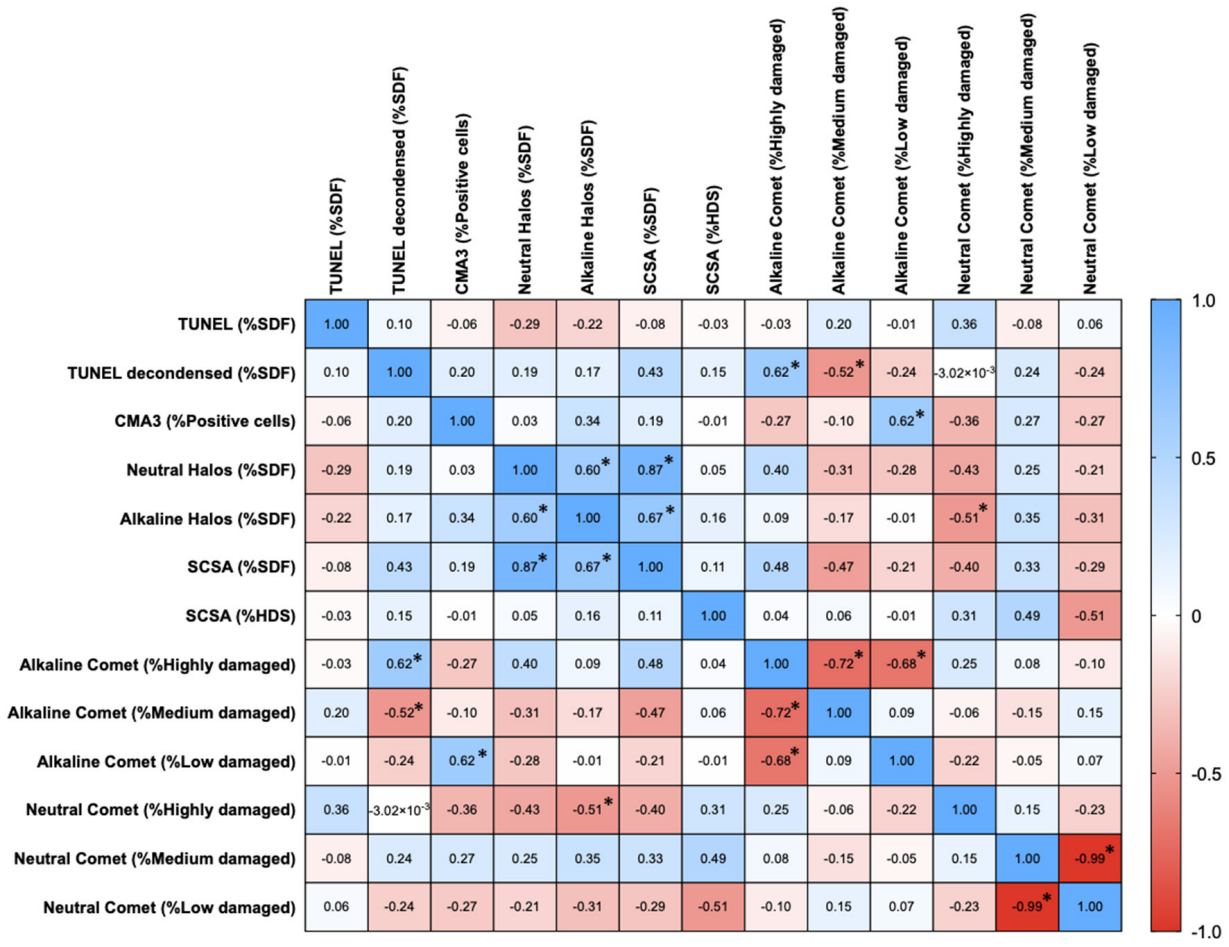
Correlation matrix between methods regarding the percentages of sperm with chromatin damage. Figures in each box represent Spearman correlation coefficients. Asterisks and bold figures indicate statistically significant correlations (*p* < 0.05).

**Figure 4:**
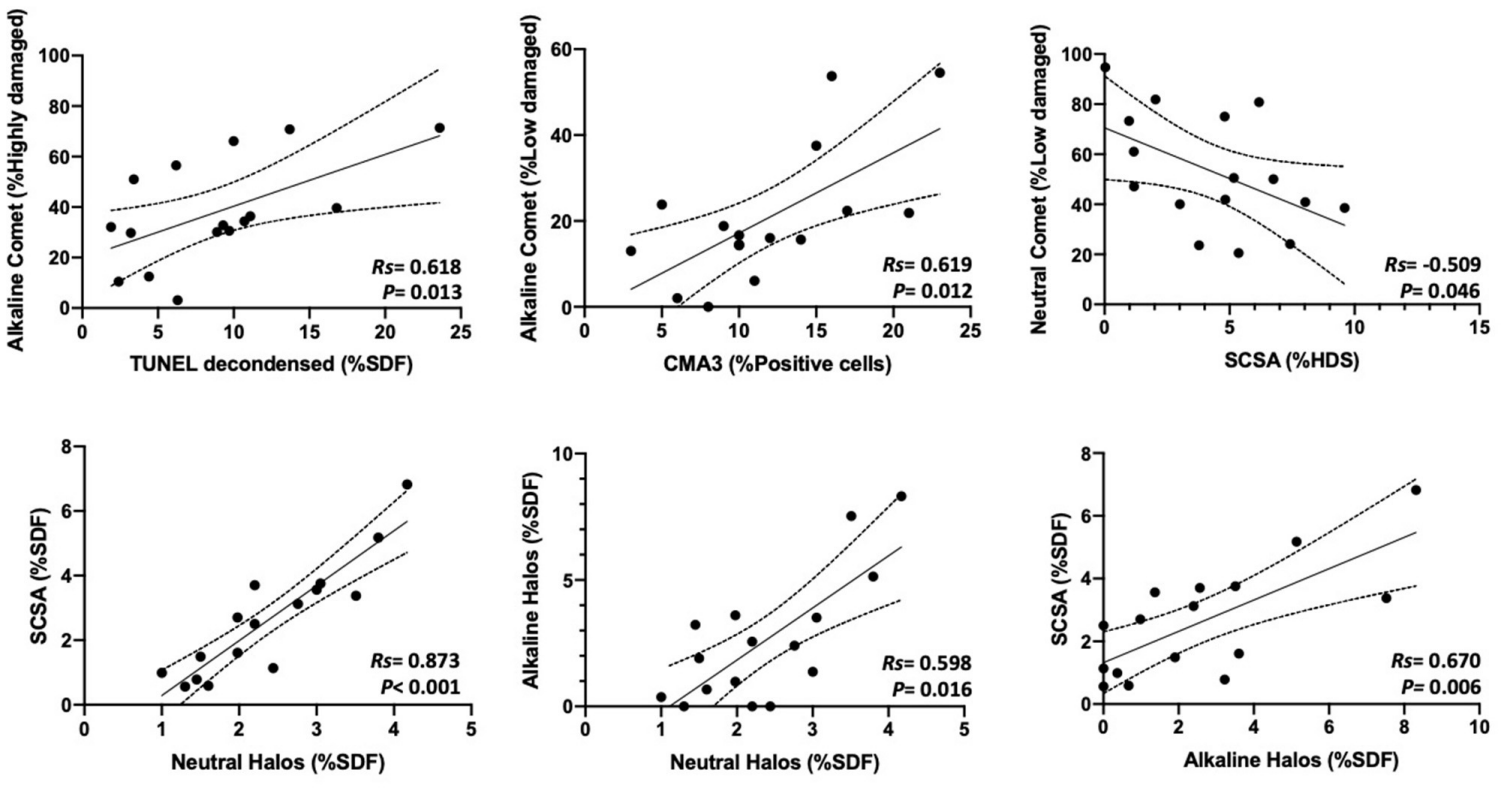
Statistically significant correlations between parameters analyzing the percentages of sperm with chromatin damage obtained through different methods.

Percentages of affected cells for each technique were measured, and correlation coefficients between methods are summarized in Figure 3. Statistically significant correlations were found between percentages of high damage in alkaline Comet and TUNEL after decondensation (*R*s = 0.618; *p* = 0.013), between percentages of low damage in alkaline Comet and CMA3 (*R*s = 0.619; *p* = 0.012), between percentages of low damage in neutral Comet and %HDS-SCSA (*R*s = −0.509; *p* = 0.046), between %SDF-SCSA and neutral SCD (*R*s = 0.873; *p* < 0.001), between alkaline and neutral SCD (*R*s = 0.598; *p* = 0.016), and between alkaline SCD and %SDF-SCSA (*R*s = 0.670; *p* = 0.006). Figure 4 shows these statistically significant correlations.

### Correlations of Chromatin Damage With Motility, Viability, and Morphology

**Figure 5:**
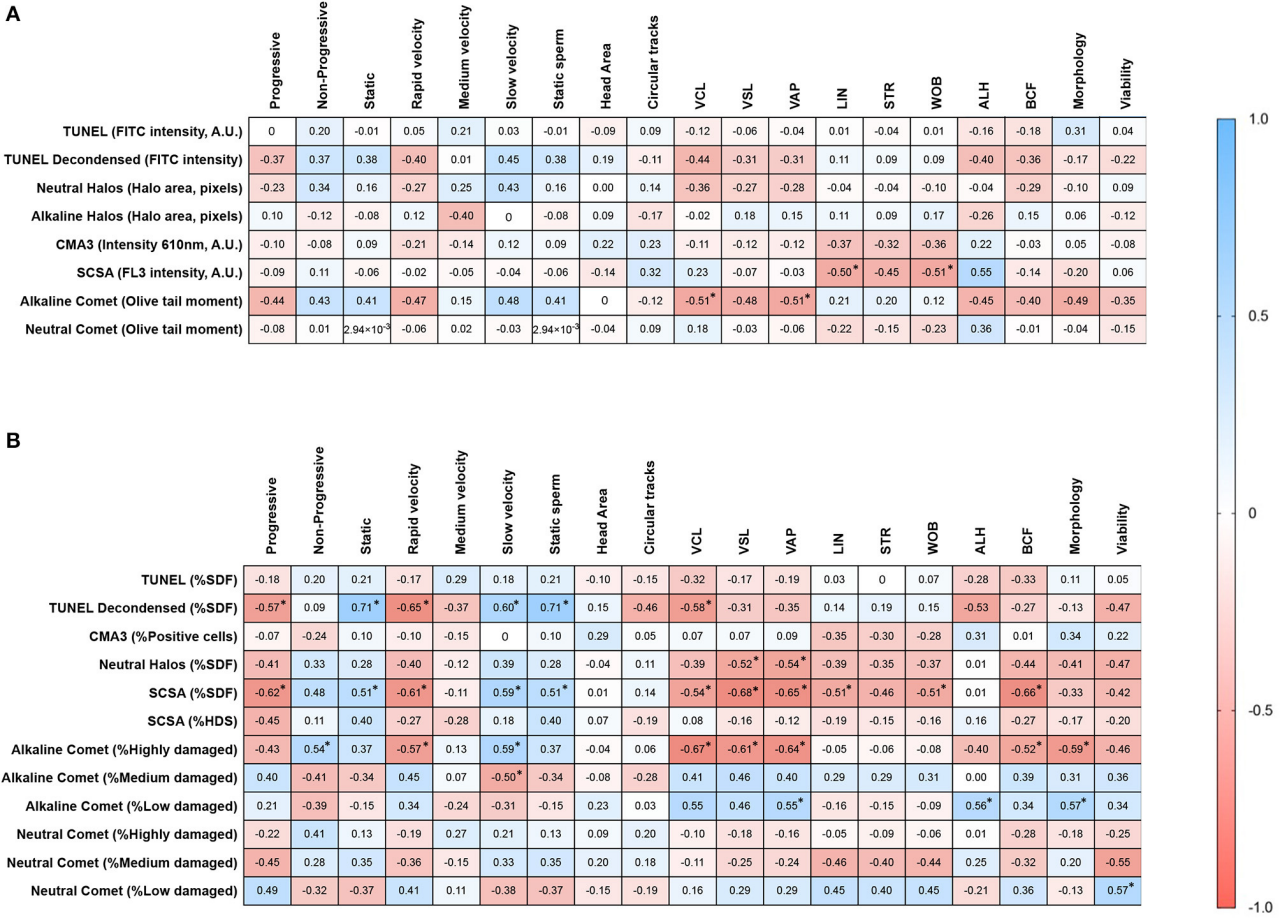
Correlation matrices of motility parameters with **(A)** the degree of chromatin damage, and **(B)** the percentages of sperm with fragmented DNA. Asterisks and bold figures indicate statistically significant correlations (*p* <0.05).

Both chromatin damage intensity and percentages of sperm with fragmented DNA were tested for their correlation with sperm motility and viability after cryopreservation and morphology. Correlation coefficients are summarized in Figure 5.

Regarding the degree of chromatin damage, statistically significant correlations between alkaline Comet OTM and VCL (*R*s = −0.514; *p* = 0.044), between SCSA-FL3 intensity and WOB (*R*s = −0.514; *p* = 0.044), and between SCSA-FL3 intensity and ALH (*R*s = 0.552; *p* = 0.029) were found. Tendencies to correlation between alkaline Comet OTM and rapid velocity (*R*s = −0.470; *p* = 0.068), alkaline Comet OTM and ALH (*R*s = −0.491; *p* = 0.055), alkaline Comet and morphology (*R*s = −0.491; *p* = 0.055), and between SCSA and LIN (*R*s = −0.500; *p* = 0.060) were observed.

With regard to the percentage of sperm with altered chromatin, statistically significant correlations were found between progressive motility and %SDF determined by TUNEL with DTT decondensation (*R*s = −0.568; *p* = 0.024); progressive motility and %SDF determined by SCSA (*R*s = −0.618; *p* = 0.013); non-progressive motility and %Highly damaged sperm in alkaline Comet (*R*s = 0.539; *p* = 0.033); sperm with rapid velocity and %SDF determined by TUNEL with DTT decondensation (*R*s = −0.653; *p* = 0.007); sperm with rapid velocity and %Highly damaged sperm in alkaline Comet (*R*s = −0.574; *p* = 0.022); VCL and %SDF determined by TUNEL with DTT decondensation (*R*s = −0.582; *p* = 0.020), %SDF determined by SCSA (*R*s = −0.544; *p* = 0.032), and %Highly damaged sperm in alkaline Comet (*R*s = −0.674; *p* = 0.005); VAP and %SDF determined by neutral SCD (*R*s = −0.536; *p* = 0.034), %SDF determined by SCSA (*R*s = −0.544; *p* = 0.008), and %Highly damaged sperm in alkaline Comet (*R*s = −0.674; *p* = 0.009).

## Discussion

In the present work, we comprehensively described the correlations among eight methodological variants assessing different facets of chromatin damage, namely, DNA breaks and poor protamination. First, our results showed that the intensity of DNA damage, given by the amount of DNA breaks, is correlated with the percentages of sperm with fragmented DNA in direct (TUNEL and Comet), but not in indirect methods (SCSA and SCD assays). Second, regarding the degree of DNA damage, we mainly found significant correlations among neutral Comet, SCSA, and conventional TUNEL; and between alkaline Comet and TUNEL with DTT decondensation. Third, as far as the percentages of sperm with altered chromatin are concerned, we observed that Comet assays, especially the alkaline variant, correlated to SCD, SCSA, and the two TUNEL variants. Interestingly, correlations between low-damaged alkaline Comet and %CMA3, and between neutral Comet and %HDS (SCSA method) were also observed.

Mounting evidence supports that sperm DNA fragmentation and alterations in sperm chromatin, such as poor protamination, underlie infertility in humans and farm animals (18, 42–46). As the sperm cell is the vehicle that brings the paternal genetic cargo into the oocyte, it seems obvious that the disruption of that material through DNA breaks may impair embryo development and reduce pregnancy rates. Indeed, sperm chromatin damage has been reported to be higher in infertile than in fertile men (45, 47–49). However, how sperm DNA damage impairs reproductive outcomes is controversial when IVF and intracytoplasmic sperm injection are compared (50–53). In this context, establishing the role of sperm DNA damage in human infertility needs more clinical data from the most sensitive and standardized DNA damage methods (26).

In production animals such as pigs, sperm quality assessment is crucial to ensure the proper performance of semen doses. While previous research with different methods evaluating sperm DNA integrity related this parameter with cryodamage in pigs, the percentages of low DNA damage led some authors to raise concerns on its biological significance (18, 54, 55). However, a few studies aiming to establish how sperm DNA damage affects fertility outcomes following artificial insemination in pigs concurred that DNA fragmentation assessed through SCSA is related to a reduction in farrowing rate and litter size (16–19). Moreover, while previous studies evaluated the repercussion of sperm DNA damage on IVF outcomes after inducing DNA breaks *in vitro* (56–58), no data regarding the inherent sperm DNA damage in untreated pig sperm samples are available. Among other reasons, the confusing results in human infertile subjects and the concerns raised from animal studies are due to the fact that no standardized method is routinely applied, and that different chromatin damage methods analyze different aspects of chromatin impairment that lead to non-comparable results (14). In the current study, and through the parallel analysis using eight techniques, we showed that the extent of chromatin damage and the percentage of altered sperm cells were correlated in direct but not in indirect methods. These findings support previous data in human sperm, in which OTM evaluated by alkaline Comet assay was observed to be related to the percentage of sperm with fragmented DNA (53). Regarding SCD, and despite previous studies having shown that this test allows evaluating the extent of chromatin damage through the halo size, which is classified as big, medium, or small (39, 59, 60), the relationship between that size and the percentage of sperm with fragmented DNA has not been explored. Moreover, while the fluorescence intensity of TUNEL, SCSA, and CMA3 may also indicate the degree of DNA/chromatin damage (30, 61), no study has investigated whether those intensities and the percentages of sperm with fragmented DNA are correlated.

To the best of our knowledge, only five studies conducted in humans and mouse compared three or more methods of chromatin evaluation using the same sperm samples (27–31). In addition, this is the first study comparing eight methodological variants for sperm chromatin assessment in pigs. On the one hand, we surprisingly observed that percentages of sperm with fragmented DNA determined by conventional TUNEL and TUNEL after DNA decondensation were not correlated. This result is of relevant importance, as TUNEL is known to be one of the most standardized methods to assess single- and double-strand DNA breaks in cells. While pre-treating sperm with 2 mM DTT increases the sensitivity of TUNEL (41), our results suggest that this is especially important in pig sperm, as their chromatin is highly impermeable and difficult to decondense (37). Remarkably, and in agreement with this hypothesis, TUNEL without decondensation did not correlate to alkaline Comet, whereas TUNEL after decondensation did (*R*s = 0.618, Figure 4).

On the other hand, percentages of sperm with low neutral Comet damage were found to be negatively correlated to %HDS evaluated through SCSA. This result confirmed a previous study conducted in mouse (31), in which a similar correlation between these two parameters was observed. Taken together, these findings suggest that the degree of chromatin decondensation could be related to double-strand DNA breaks. In this regard, one could hypothesize that when sperm are morphologically immature, which is linked to a higher number of histones retained and highly stainable DNA (%HDS), enzymes that like nucleases perform double-strand breaks can easily access chromatin and thus cause DNA breaks in non-protaminated regions. In fact, the presence of internal nucleases, which cause chromatin damage and have been described to exist in human, mouse, and hamster sperm, supports this hypothesis (62, 63).

Percentages of sperm with intact DNA determined by alkaline Comet were correlated with those of CMA3^+^ sperm. At first glance, this result could seem contradictory, as CMA3 is known to be a marker of abnormal protamination (64, 65). However, it has been previously described that GC-rich sequences are preferential binding sites for CMA3 (66). This is of vital importance, since the oxidized form of guanine (8-hydroxyguanine) is the main target of oxidative DNA damage, which can also be measured through the alkaline Comet (20, 67). Therefore, this observed correlation could be explained by the presence of DNA breaks in guanine-rich sequences, which could lead to less CMA3 binding. In addition, it is worth mentioning that while the percentages of sperm with fragmented DNA correlated between indirect methods (SCD and SCSA), they did not appear to be associated to those of CMA3^+^ sperm. This suggests that, despite SCD and SCSA relying on chromatin decondensation as a measure of DNA fragmentation, they are not related to abnormal chromatin protamination.

Finally, we investigated whether the different methods assessing DNA damage were associated to motility parameters. We observed that direct methods such as TUNEL and alkaline Comet, and indirect ones like SCSA, were correlated with progressive motility and sperm velocity. Previous studies in humans also reported that association (68–70), which may represent a positive bias for ICSI treatments, as the most motile sperm are selected (71).

The present study is not exempt of limitations. First, despite the fact that pig sperm samples exhibit higher homogeneity than their human counterparts, thus adding robustness to our work, further research using larger sample sizes for each method is needed. Second, future studies should analyze DNA/chromatin damage in fresh and cryopreserved sperm, as freezing and thawing may affect DNA integrity (72). Finally, intra- and inter-assay variations should be determined in order to define the most robust method to assess sperm DNA damage.

In conclusion, the current study indicates that while the extent of chromatin damage is correlated with the percentage of sperm with fragmented DNA determined by direct methods (alkaline and neutral Comet, and TUNEL following decondensation), this is not the case of indirect methods (SCD and SCSA). In addition, in pig sperm, previous decondensation of 2 mM DTT is required in order for TUNEL assay to show reliable levels of sperm DNA fragmentation. Thus, direct rather than indirect methods are suggested to be more suitable to evaluate DNA fragmentation in pig sperm.

## Data Availability Statement

The raw data supporting the conclusions of this article will be made available by the authors, without undue reservation.

## Author Contributions

JR-M conceived the study, performed DNA damage experiments, analyzed data, conducted statistics, wrote the manuscript, revised the document, and approved the final version. ML, YM-O, and AD-B conducted CMA3 and viability experiments, discussed the results, and approved the final version. EG-B performed cryopreservation, evaluated sperm motility and morphology, and approved the final version. MY contributed to the experimental design, provided funding, coordinated the work, made a critical revision of the manuscript, and approved the final version. All authors contributed to the article and approved the submitted version.

## Conflict of Interest

The authors declare that the research was conducted in the absence of any commercial or financial relationships that could be construed as a potential conflict of interest.

## Publisher's Note

All claims expressed in this article are solely those of the authors and do not necessarily represent those of their affiliated organizations, or those of the publisher, the editors and the reviewers. Any product that may be evaluated in this article, or claim that may be made by its manufacturer, is not guaranteed or endorsed by the publisher.

## Supplementary Material

Supplementary Figure 1 Representative phase-contrast and epifluorescence images of negative and positive controls (incubation with 5 mM DTT at 37°C for 45 min) used to set up the CMA3 method. Scale bar = XX μm.

Supplementary Figure 2 Representative phase-contrast and epifluorescence images of negative and positive controls (incubation with 4 IU/μL DNAse I at 37°C for 1 h) used to set up the TUNEL method. Scale bar = XX μm.

Supplementary Table 1 Data including correlations and *P*-values for the degree of sperm chromatin damage, as depicted in Figure 1.

Supplementary Table 2 Data including correlations and *P*-values for the percentages of sperm with chromatin damage, as shown in Figure 3.

Supplementary Table 3 Data including correlations and *P*-values of sperm motility with **(A)** chromatin damage intensity, and **(B)** percentage of cells with chromatin damage, as depicted in Figure 5.
