# Supplementary figures and images for: Direct but Not Indirect Methods Correlate the Percentages of Sperm With Altered Chromatin to the Intensity of Chromatin Damage
Source: Front Vet Sci. 2021 Aug 25;8:719319. doi: 10.3389/fvets.2021.719319 (PMC8570191; doi:10.3389/fvets.2021.719319)

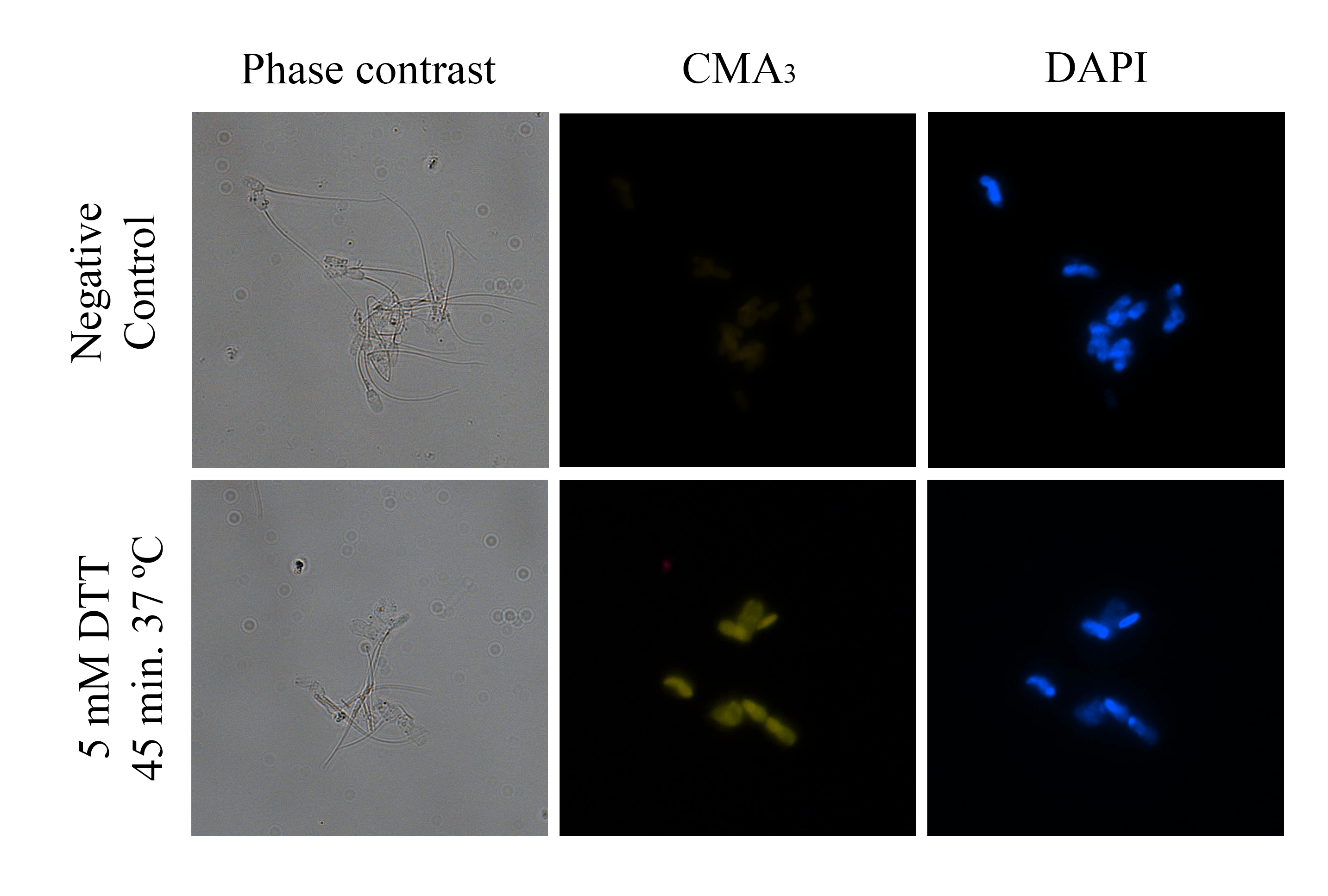

Supplement: Supplementary Figure 1 — Representative phase-contrast and epifluorescence images of negative and positive controls (incubation with 5 mM DTT at 37°C for 45 min) used to set up the CMA3 method. Scale bar = XX μm. [file Image_1.tif]

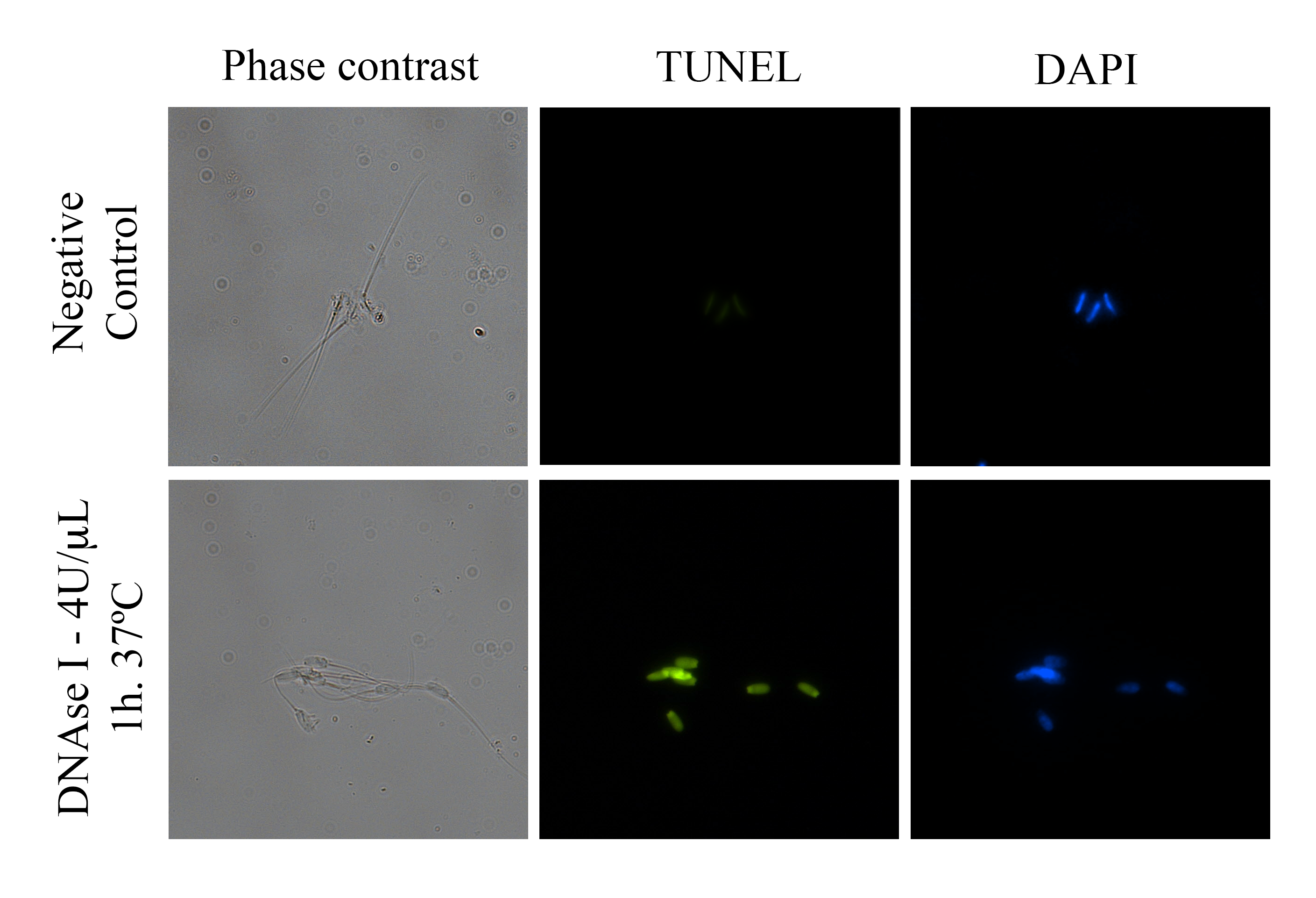

Supplement: Supplementary Figure 2 — Representative phase-contrast and epifluorescence images of negative and positive controls (incubation with 4 IU/μL DNAse I at 37°C for 1 h) used to set up the TUNEL method. Scale bar = XX μm. [file Image_2.tif]
