# Supplementary material for: Direct but Not Indirect Methods Correlate the Percentages of Sperm With Altered Chromatin to the Intensity of Chromatin Damage
Source: Front Vet Sci. 2021 Aug 25;8:719319. doi: 10.3389/fvets.2021.719319 (PMC8570191; doi:10.3389/fvets.2021.719319)
Supplement: Supplementary Table 1 — Data including correlations and P-values for the degree of sperm chromatin damage, as depicted in Figure 1. [file Table_1.docx]

**Supplementary Table 1.** Data including correlations and *P*-values for the degree of sperm chromatin damage, as depicted in Figure 1.

|  |  | TUNEL  (FITC intensity, A.U.) | | |  |  |  |  |
| --- | --- | --- | --- | --- | --- | --- | --- | --- |
| TUNEL decondensed  (FITC intensity, A.U.) | Rs | 0,335 |  |  |  |  |  |  |
|  | *P*-value | 0,204 | TUNEL decondensed (FITC intensity, A.U.) | | | |  |  |
| CMA3  (Intensity 610nm, A.U.) | Rs | **-0,579** | -0,353 |  |  |  |  |  |
|  | *P*-value | **0,021** | 0,180 | CMA3 (Intensity 610nm, A.U.) | | |  |  |
| Neutral Halos  (Halo area, pixels) | Rs | 0,097 | 0,288 | 0,194 |  |  |  |  |
|  | *P*-value | 0,721 | 0,278 | 0,470 | Neutral Halos (Halo area, pixels) | | |  |
| Alkaline Halos (Halo area, pixels) | Rs | 0,288 | 0,462 | -0,126 | 0,035 |  |  |  |
|  | *P*-value | 0,278 | 0,074 | 0,641 | 0,900 | Alkaline Halos (Halo area, pixels) | | |
| SCSA (FL3 intensity, A.U.) | Rs | -0,429 | -0,421 | 0,221 | -0,188 | -0,297 |  |  |
|  | *P*-value | 0,099 | 0,106 | 0,410 | 0,484 | 0,263 | SCSA (FL3 intensity, A.U.) | |
| Alkaline Comet (Olive tail moment) | Rs | -0,018 | **0,741** | -0,326 | 0,244 | 0,021 | -0,306 |  |
|  | *P*-value | 0,952 | **0,001** | 0,217 | 0,361 | 0,943 | 0,249 | Alkaline Comet (Olive tail moment) |
| Neutral Comet (Olive tail moment) | Rs | **-0,535** | -0,409 | 0,274 | -0,444 | -0,421 | **0,668** | -0,062 |
|  | *P*-value | **0,035** | 0,117 | 0,304 | 0,087 | 0,106 | **0,006** | 0,822 |
