# Supplementary material for: Direct but Not Indirect Methods Correlate the Percentages of Sperm With Altered Chromatin to the Intensity of Chromatin Damage
Source: Front Vet Sci. 2021 Aug 25;8:719319. doi: 10.3389/fvets.2021.719319 (PMC8570191; doi:10.3389/fvets.2021.719319)
Supplement: Supplementary Table 2 — Data including correlations and P-values for the percentages of sperm with chromatin damage, as shown in Figure 3. [file Table_2.docx]

**Supplementary Table 2.** Data including correlations and *P*-values for the percentages of sperm with chromatin damage, as shown in Figure 3.

|  |  | TUNEL (%SDF) | |  |  |  |  |  |  |  |  |  |  |
| --- | --- | --- | --- | --- | --- | --- | --- | --- | --- | --- | --- | --- | --- |
| TUNEL decondensed (%SDF) | Rs | 0,096 |  |  |  |  |  |  |  |  |  |  |  |
|  | *P*-value | 0,722 | TUNEL decondensed (%SDF) | |  |  |  |  |  |  |  |  |  |
| CMA3 (%Positive cells) | Rs | -0,062 | 0,198 |  |  |  |  |  |  |  |  |  |  |
|  | *P*-value | 0,819 | 0,462 | CMA3 (%Positive cells) | |  |  |  |  |  |  |  |  |
| Neutral Halos (%SDF) | Rs | -0,292 | 0,187 | 0,032 |  |  |  |  |  |  |  |  |  |
|  | *P*-value | 0,270 | 0,485 | 0,906 | Neutral Halos (%SDF) | |  |  |  |  |  |  |  |
| Alkaline Halos (%SDF) | Rs | -0,216 | 0,168 | 0,340 | **0,598** |  |  |  |  |  |  |  |  |
|  | *P*-value | 0,417 | 0,532 | 0,197 | **0,016** | Alkaline Halos (%SDF) | |  |  |  |  |  |  |
| SCSA (%SDF) | Rs | -0,075 | 0,426 | 0,186 | **0,873** | **0,670** |  |  |  |  |  |  |  |
|  | *P*-value | 0,781 | 0,101 | 0,490 | **0,000** | **0,006** | SCSA (%SDF) |  |  |  |  |  |  |
| SCSA (%HDS) | Rs | -0,027 | 0,153 | -0,006 | 0,052 | 0,162 | 0,115 |  |  |  |  |  |  |
|  | *P*-value | 0,923 | 0,571 | 0,987 | 0,850 | 0,547 | 0,672 | SCSA (%HDS) |  |  |  |  |  |
| Alkaline Comet (%Highly damaged) | Rs | -0,030 | **0,618** | -0,274 | 0,398 | 0,086 | 0,476 | 0,035 |  |  |  |  |  |
|  | *P*-value | 0,914 | **0,013** | 0,303 | 0,128 | 0,754 | 0,064 | 0,900 | Alkaline Comet (%Highly damaged) | | |  |  |
| Alkaline Comet (%Medium damaged) | Rs | 0,198 | **-0,519** | -0,103 | -0,312 | -0,174 | -0,474 | 0,063 | **-0,715** |  |  |  |  |
|  | *P*-value | 0,458 | **0,041** | 0,702 | 0,238 | 0,516 | 0,065 | 0,816 | **0,002** | Alkaline Comet (%Medium damaged) | | |  |
| Alkaline Comet (%Low damaged) | Rs | -0,006 | -0,241 | **0,619** | -0,278 | -0,009 | -0,212 | -0,009 | **-0,682** | 0,094 |  |  |  |
|  | *P*-value | 0,985 | 0,367 | **0,012** | 0,294 | 0,978 | 0,430 | 0,978 | **0,005** | 0,727 | Alkaline Comet (%Low damaged) | | |
| Neutral Comet (%Highly damaged) | Rs | 0,361 | -0,003 | -0,362 | -0,434 | **-0,506** | -0,405 | 0,308 | 0,246 | -0,060 | -0,222 |  |  |
|  | *P*-value | 0,168 | 0,993 | 0,167 | 0,094 | **0,048** | 0,120 | 0,243 | 0,354 | 0,825 | 0,404 | Neutral Comet (%Highly damaged) | |
| Neutral Comet (%Medium damaged) | Rs | -0,083 | 0,241 | 0,265 | 0,247 | 0,348 | 0,329 | 0,488 | 0,082 | -0,152 | -0,047 | 0,145 |  |
|  | *P*-value | 0,760 | 0,367 | 0,319 | 0,353 | 0,187 | 0,213 | 0,057 | 0,763 | 0,573 | 0,865 | 0,589 | Neutral Comet (%Medium damaged) |
| Neutral Comet (%Low damaged) | Rs | 0,056 | -0,241 | -0,265 | -0,209 | -0,313 | -0,291 | -0,509 | -0,100 | 0,150 | 0,068 | -0,227 | **-0,991** |
|  | *P*-value | 0,836 | 0,367 | 0,319 | 0,434 | 0,238 | 0,273 | 0,046 | 0,713 | 0,576 | 0,805 | 0,395 | **0,000** |
