# Supplementary material for: Direct but Not Indirect Methods Correlate the Percentages of Sperm With Altered Chromatin to the Intensity of Chromatin Damage
Source: Front Vet Sci. 2021 Aug 25;8:719319. doi: 10.3389/fvets.2021.719319 (PMC8570191; doi:10.3389/fvets.2021.719319)
Supplement: Supplementary Table 3 — Data including correlations and P-values of sperm motility with (A) chromatin damage intensity, and (B) percentage of cells with chromatin damage, as depicted in Figure 5. [file Table_3.docx]

**Supplementary Table 3.** Data including correlations and *P*-values of sperm motility with (A) chromatin damage intensity, and (B) percentage of cells with chromatin damage, as depicted in Figure 5.

| A |  |  |  |  |  |  |  |  |  |  |  |  |  |  |  |  |  |  |  |  |
| --- | --- | --- | --- | --- | --- | --- | --- | --- | --- | --- | --- | --- | --- | --- | --- | --- | --- | --- | --- | --- |
|  |  | Progressive | Non-Progressive | Static | Rapid velocity | Medium velocity | Slow velocity | Static sperm | Head Area | Circular tracks | VCL | VSL | VAP | LIN | STR | WOB | ALH | BCF | Morphology | Viability |
| TUNEL (FITC intensity, A.U.) | Rs | -0,003 | 0,201 | -0,015 | 0,053 | 0,208 | 0,034 | -0,015 | -0,091 | 0,088 | -0,118 | -0,059 | -0,035 | 0,006 | -0,038 | 0,012 | -0,156 | -0,176 | 0,311 | 0,044 |
|  | *P*-value | 0,996 | 0,456 | 0,957 | 0,846 | 0,439 | 0,901 | 0,957 | 0,737 | 0,745 | 0,664 | 0,829 | 0,897 | 0,983 | 0,888 | 0,966 | 0,564 | 0,513 | 0,242 | 0,871 |
| TUNEL Decondensed (FITC intensity) | Rs | -0,368 | 0,375 | 0,379 | -0,397 | 0,013 | 0,449 | 0,379 | 0,188 | -0,109 | -0,435 | -0,309 | -0,309 | 0,115 | 0,094 | 0,088 | -0,397 | -0,365 | -0,172 | -0,221 |
|  | *P*-value | 0,162 | 0,152 | 0,147 | 0,128 | 0,961 | 0,081 | 0,147 | 0,485 | 0,688 | 0,092 | 0,244 | 0,244 | 0,672 | 0,729 | 0,745 | 0,128 | 0,165 | 0,524 | 0,412 |
| Neutral Halos (Halo area, pixels) | Rs | -0,226 | 0,342 | 0,162 | -0,268 | 0,248 | 0,433 | 0,162 | 0,000 | 0,141 | -0,365 | -0,271 | -0,282 | -0,035 | -0,041 | -0,097 | -0,035 | -0,291 | -0,104 | 0,085 |
|  | *P*-value | 0,398 | 0,194 | 0,549 | 0,316 | 0,354 | 0,094 | 0,549 | 1,000 | 0,602 | 0,165 | 0,311 | 0,289 | 0,897 | 0,880 | 0,721 | 0,897 | 0,274 | 0,700 | 0,753 |
| Alkaline Halos (Halo area, pixels) | Rs | 0,097 | -0,120 | -0,079 | 0,121 | -0,400 | -0,003 | -0,079 | 0,094 | -0,174 | -0,024 | 0,176 | 0,150 | 0,106 | 0,088 | 0,174 | -0,256 | 0,153 | 0,065 | -0,121 |
|  | *P*-value | 0,721 | 0,659 | 0,770 | 0,656 | 0,124 | 0,991 | 0,770 | 0,729 | 0,520 | 0,931 | 0,513 | 0,579 | 0,696 | 0,745 | 0,520 | 0,339 | 0,572 | 0,812 | 0,656 |
| CMA3 (Intensity 610nm, A.U.) | Rs | -0,103 | -0,077 | 0,088 | -0,215 | -0,137 | 0,124 | 0,088 | 0,224 | 0,232 | -0,106 | -0,124 | -0,118 | -0,371 | -0,324 | -0,365 | 0,218 | -0,029 | 0,054 | -0,076 |
|  | *P*-value | 0,705 | 0,778 | 0,745 | 0,425 | 0,612 | 0,647 | 0,745 | 0,405 | 0,387 | 0,696 | 0,649 | 0,664 | 0,158 | 0,222 | 0,165 | 0,418 | 0,914 | 0,841 | 0,778 |
| SCSA (FL3 intensity, A.U.) | Rs | -0,088 | 0,114 | -0,062 | -0,018 | -0,050 | -0,043 | -0,062 | -0,144 | 0,321 | 0,229 | -0,074 | -0,032 | **-0,500** | -0,450 | **-0,515** | 0,553 | -0,138 | -0,202 | 0,065 |
|  | *P*-value | 0,746 | 0,675 | 0,820 | 0,948 | 0,853 | 0,875 | 0,820 | 0,594 | 0,226 | 0,393 | 0,787 | 0,905 | **0,049** | 0,080 | **0,041** | 0,026 | 0,610 | 0,454 | 0,812 |
| Alkaline Comet (Olive tail moment) | Rs | -0,438 | 0,428 | 0,415 | -0,471 | 0,146 | 0,483 | 0,415 | -0,003 | -0,124 | **-0,515** | -0,476 | **-0,515** | 0,206 | 0,197 | 0,121 | -0,453 | -0,403 | -0,492 | -0,347 |
|  | *P*-value | 0,091 | 0,098 | 0,110 | 0,066 | 0,589 | 0,058 | 0,110 | 0,991 | 0,649 | **0,041** | 0,062 | **0,041** | 0,444 | 0,464 | 0,656 | 0,078 | 0,122 | 0,053 | 0,188 |
| Neutral Comet (Olive tail moment) | Rs | -0,082 | 0,012 | 0,003 | -0,056 | 0,024 | -0,034 | 0,003 | -0,044 | 0,091 | 0,182 | -0,032 | -0,059 | -0,218 | -0,153 | -0,232 | 0,362 | -0,012 | -0,037 | -0,147 |
|  | *P*-value | 0,763 | 0,965 | 0,991 | 0,837 | 0,931 | 0,901 | 0,991 | 0,871 | 0,737 | 0,499 | 0,905 | 0,829 | 0,418 | 0,572 | 0,387 | 0,169 | 0,966 | 0,892 | 0,587 |
|  |  |  |  |  |  |  |  |  |  |  |  |  |  |  |  |  |  |  |  |  |
| B |  |  |  |  |  |  |  |  |  |  |  |  |  |  |  |  |  |  |  |  |
|  |  | Progressive | Non-Progressive | Static | Rapid velocity | Medium velocity | Slow velocity | Static sperm | Head Area | Circular tracks | VCL | VSL | VAP | LIN | STR | WOB | ALH | BCF | Morphology | Viability |
| TUNEL (%SDF) | Rs | -0,176 | 0,201 | 0,210 | -0,174 | 0,287 | 0,185 | 0,210 | -0,101 | -0,152 | -0,322 | -0,166 | -0,191 | 0,033 | -0,001 | 0,069 | -0,276 | -0,330 | 0,109 | 0,050 |
|  | *P*-value | 0,512 | 0,455 | 0,435 | 0,518 | 0,281 | 0,493 | 0,435 | 0,711 | 0,573 | 0,224 | 0,540 | 0,479 | 0,905 | 0,996 | 0,798 | 0,300 | 0,212 | 0,689 | 0,853 |
| TUNEL Decondensed (%SDF) | Rs | **-0,568** | 0,094 | **0,715** | **-0,653** | -0,366 | **0,604** | **0,715** | 0,150 | -0,465 | **-0,582** | -0,309 | -0,347 | 0,144 | 0,185 | 0,153 | -0,532 | -0,274 | -0,130 | -0,471 |
|  | *P*-value | **0,024** | 0,728 | **0,002** | **0,006** | 0,163 | **0,013** | **0,002** | 0,579 | 0,070 | **0,018** | 0,244 | 0,188 | 0,594 | 0,492 | 0,572 | 0,034 | 0,305 | 0,633 | 0,066 |
| CMA3 (%Positive cells) | Rs | -0,068 | -0,237 | 0,100 | -0,103 | -0,147 | -0,003 | 0,100 | 0,295 | 0,047 | 0,071 | 0,074 | 0,091 | -0,345 | -0,298 | -0,283 | 0,307 | 0,012 | 0,335 | 0,224 |
|  | *P*-value | 0,805 | 0,377 | 0,712 | 0,704 | 0,588 | 0,991 | 0,712 | 0,267 | 0,862 | 0,794 | 0,786 | 0,736 | 0,190 | 0,262 | 0,288 | 0,248 | 0,965 | 0,205 | 0,404 |
| Neutral Halos (%SDF) | Rs | -0,409 | 0,335 | 0,283 | -0,402 | -0,115 | 0,389 | 0,283 | -0,040 | 0,113 | -0,387 | **-0,524** | **-0,536** | -0,390 | -0,355 | -0,374 | 0,012 | -0,443 | -0,413 | -0,470 |
|  | *P*-value | 0,116 | 0,205 | 0,289 | 0,123 | 0,670 | 0,136 | 0,289 | 0,884 | 0,676 | 0,138 | **0,037** | **0,032** | 0,135 | 0,177 | 0,153 | 0,965 | 0,085 | 0,112 | 0,066 |
| SCSA (%SDF) | Rs | **-0,618** | 0,480 | **0,506** | **-0,615** | -0,106 | **0,594** | **0,506** | 0,015 | 0,141 | **-0,544** | **-0,676** | **-0,650** | **-0,509** | -0,462 | **-0,506** | 0,009 | **-0,662** | -0,328 | -0,424 |
|  | *P*-value | **0,013** | 0,060 | **0,046** | **0,011** | 0,695 | **0,015** | **0,046** | 0,957 | 0,602 | **0,029** | **0,004** | **0,006** | **0,044** | 0,072 | **0,046** | 0,974 | **0,005** | 0,215 | 0,102 |
| SCSA (%HDS) | Rs | -0,453 | 0,106 | 0,400 | -0,274 | -0,281 | 0,177 | 0,400 | 0,071 | -0,188 | 0,076 | -0,156 | -0,124 | -0,194 | -0,150 | -0,162 | 0,162 | -0,271 | -0,171 | -0,200 |
|  | *P*-value | 0,080 | 0,695 | 0,125 | 0,305 | 0,292 | 0,511 | 0,125 | 0,795 | 0,485 | 0,778 | 0,564 | 0,649 | 0,471 | 0,579 | 0,549 | 0,549 | 0,311 | 0,527 | 0,458 |
| Alkaline Comet (%Highly damaged) | Rs | -0,429 | **0,539** | 0,371 | **-0,574** | 0,129 | **0,591** | 0,371 | -0,035 | 0,065 | **-0,674** | **-0,609** | **-0,638** | -0,050 | -0,062 | -0,076 | -0,400 | **-0,524** | **-0,593** | -0,456 |
|  | *P*-value | 0,099 | **0,031** | 0,158 | **0,020** | 0,635 | **0,016** | 0,158 | 0,897 | 0,812 | **0,004** | **0,012** | **0,008** | 0,854 | 0,820 | 0,778 | 0,125 | **0,037** | **0,015** | 0,076 |
| Alkaline Comet (%Medium damaged) | Rs | 0,397 | -0,405 | -0,336 | 0,447 | 0,065 | **-0,503** | -0,336 | -0,078 | -0,275 | 0,414 | 0,464 | 0,399 | 0,286 | 0,286 | 0,312 | 0,000 | 0,387 | 0,306 | 0,359 |
|  | *P*-value | 0,128 | 0,119 | 0,204 | 0,082 | 0,811 | **0,047** | 0,204 | 0,774 | 0,302 | 0,111 | 0,071 | 0,126 | 0,284 | 0,284 | 0,239 | 1,000 | 0,139 | 0,249 | 0,172 |
| Alkaline Comet (%Low damaged) | Rs | 0,206 | -0,387 | -0,150 | 0,335 | -0,239 | -0,313 | -0,150 | 0,229 | 0,032 | 0,553 | 0,456 | **0,553** | -0,159 | -0,147 | -0,085 | **0,556** | 0,341 | **0,572** | 0,335 |
|  | *P*-value | 0,443 | 0,139 | 0,579 | 0,204 | 0,372 | 0,238 | 0,579 | 0,393 | 0,905 | 0,026 | 0,076 | **0,026** | 0,557 | 0,587 | 0,753 | **0,025** | 0,196 | **0,020** | 0,204 |
| Neutral Comet (%Highly damaged) | Rs | -0,216 | 0,413 | 0,130 | -0,193 | 0,268 | 0,209 | 0,130 | 0,089 | 0,198 | -0,095 | -0,180 | -0,160 | -0,048 | -0,094 | -0,062 | 0,009 | -0,281 | -0,175 | -0,248 |
|  | *P*-value | 0,418 | 0,112 | 0,631 | 0,473 | 0,316 | 0,436 | 0,631 | 0,743 | 0,462 | 0,726 | 0,505 | 0,553 | 0,859 | 0,730 | 0,820 | 0,973 | 0,292 | 0,516 | 0,355 |
| Neutral Comet (%Medium damaged) | Rs | -0,450 | 0,279 | 0,347 | -0,356 | -0,146 | 0,328 | 0,347 | 0,197 | 0,182 | -0,109 | -0,247 | -0,238 | -0,456 | -0,403 | -0,444 | 0,247 | -0,318 | 0,199 | -0,553 |
|  | *P*-value | 0,082 | 0,295 | 0,188 | 0,176 | 0,589 | 0,215 | 0,188 | 0,464 | 0,499 | 0,688 | 0,356 | 0,374 | 0,076 | 0,122 | 0,085 | 0,356 | 0,231 | 0,461 | 0,026 |
| Neutral Comet (%Low damaged) | Rs | 0,485 | -0,320 | -0,374 | 0,412 | 0,114 | -0,377 | -0,374 | -0,150 | -0,191 | 0,162 | 0,294 | 0,294 | 0,453 | 0,400 | 0,447 | -0,215 | 0,359 | -0,127 | **0,568** |
|  | *P*-value | 0,059 | 0,226 | 0,154 | 0,113 | 0,675 | 0,150 | 0,154 | 0,579 | 0,478 | 0,549 | 0,269 | 0,269 | 0,078 | 0,125 | 0,083 | 0,425 | 0,172 | 0,640 | **0,022** |
